# The effect of different vitrification protocols on cell survival in human ovarian tissue: a pilot study

**DOI:** 10.1186/s13048-021-00924-8

**Published:** 2021-12-06

**Authors:** J. Marschalek, C. Egarter, K. Nouri, S. Dekan, J. Ott, M. Frank, D. Pietrowski

**Affiliations:** 1 Department of Obstetrics and Gynecology, Medical University of Vienna, Spitalgasse 23, 1090 Vienna, Austria; 2 Clinical Institute of Pathology, Medical University of Vienna, Spitalgasse 230, 1090 Vienna, Austria

**Keywords:** Vitrification, Fertility preservation, Ovarian tissue cryopreservation, Cell viability, Transgender

## Abstract

**Background:**

Vitrification has superseded the slow freezing method for cryopreservation of oocytes, embryos, and sperm, but there are as yet no standard protocols for its use in ovarian tissue cryopreservation (OTC). Published protocols diverge mainly with regard to the extent of supplementation of dimethyl sulfoxide (DMSO) to the vitrification medium, and to the use of an open or closed vitrification system.

We investigated the viability of cells after vitrification/warming, using ovarian tissue of transgender patients, by means of Fluorescence Activated Cells Sorting (FACS), and histomorphological analyses using a DMSO-containing (P1) and a DMSO-free protocol (P2) in an open or closed vitrification setting.

**Results:**

Twelve ovarian samples were donated from female-to-male transgender patients: 6 were vitrified according to protocol 1, the other 6 according to protocol 2. The amount of viable cells was 90.1% (P1) and 88.4% (P2) before vitrification. After vitrification and subsequent warming, viable cells were reduced to 82.9% (P1, *p* = 0.093) and 72.4% (P2, *p* = 0.019). When comparing the closed and the open systems, the decline in cell viability from pre- to post-vitrification was significant only for the latter (*p =* 0.037). Histological examination reveals no significant differences with respect to degenerated follicles before or after vitrification.

**Conclusion:**

These results led us to conclude that a protocol containing DMSO results in a higher viability of ovarian cells than a protocol that uses ethylene glycol as cryoprotective agent in vitrification. The use of an open vitrification system led to significant decline in the rate of viable cells.

**Trial registration:**

NCT03649087, retrospectively registered 28.08.2018.

## Introduction

Ovarian tissue cryopreservation (OTC) is reported to be a successful way to preserve the fertility in women undergoing sterilizing cancer therapy [1, 2] and the interest in its re-transplantation is rapidly growing, with more than 200 live births reported to date [3, 4]. Although the majority of these live births were achieved with the slow freezing method [5, 6], live births were also reported following vitrification of ovarian tissue, which represents an ultra-fast freezing procedure by direct immersion of the tissue in liquid nitrogen [1, 7]. The literature, however, provides conflicting results with respect to the outcomes after cryopreservation of ovarian tissue with vitrification, as opposed to the slow freezing method, which to date serves as the standard method [6, 8].

In OTC, it is vital to retain the complex nature of ovarian tissue with its variety of cell types in order to restore the ovarian function after re-transplantation. Studies evaluating the slow freezing method reported negative effects on ovarian tissues, postulating that different cell types in ovarian tissue require different factors to prevent damage from ice crystal formation [9] Moreover, Sheikhi and colleagues postulated that vitrification could be advantageous over the slow freezing method, as a result of it not inducing apoptosis in mouse and human ovarian tissue after warming [10]. Presupposing that the tissue is small enough to assure rapid cooling during vitrification, cell-damaging ice crystal formation can be avoided. Vitrification is routinely used in assisted reproduction for cryopreservation of oocytes, embryos and sperm - which are very small compared to an ovarian tissue segment. OTC and re-transplantation is still experimental and published vitrification-protocols diverge mainly on the issue of supplementation of dimethyl sulfoxide (DMSO) to the vitrification media on the one hand, and the use of an open or closed vitrification system on the other [1, 11]. With a direct exposure of the ovarian tissue to liquid nitrogen, an open vitrification system bears the risk of a contamination with pathogens. Hence, open vitrification systems are viewed critically and some authors recommend the scientifically proven success of the closed vitrification system [12, 13].

In general, the chemicals used in cryoprotective solutions are considered to be toxic to various cells. The degree of toxicity is dependent on the cell type and on the concentration of the cryoprotectant. Therefore, the current difficulty is to identify cryoprotective agents that do not affect cell viability and which can be used for vitrification in acceptable concentrations at feasible cooling and warming rates.

As protocols for vitrification in OTC vary from center to center, we aimed to study the impact of vitrification on human ovarian tissue by comparing the effects of two solutions either in- or excluding DMSO in an open and closed vitrification system.

## Results

**Fig. 1:**
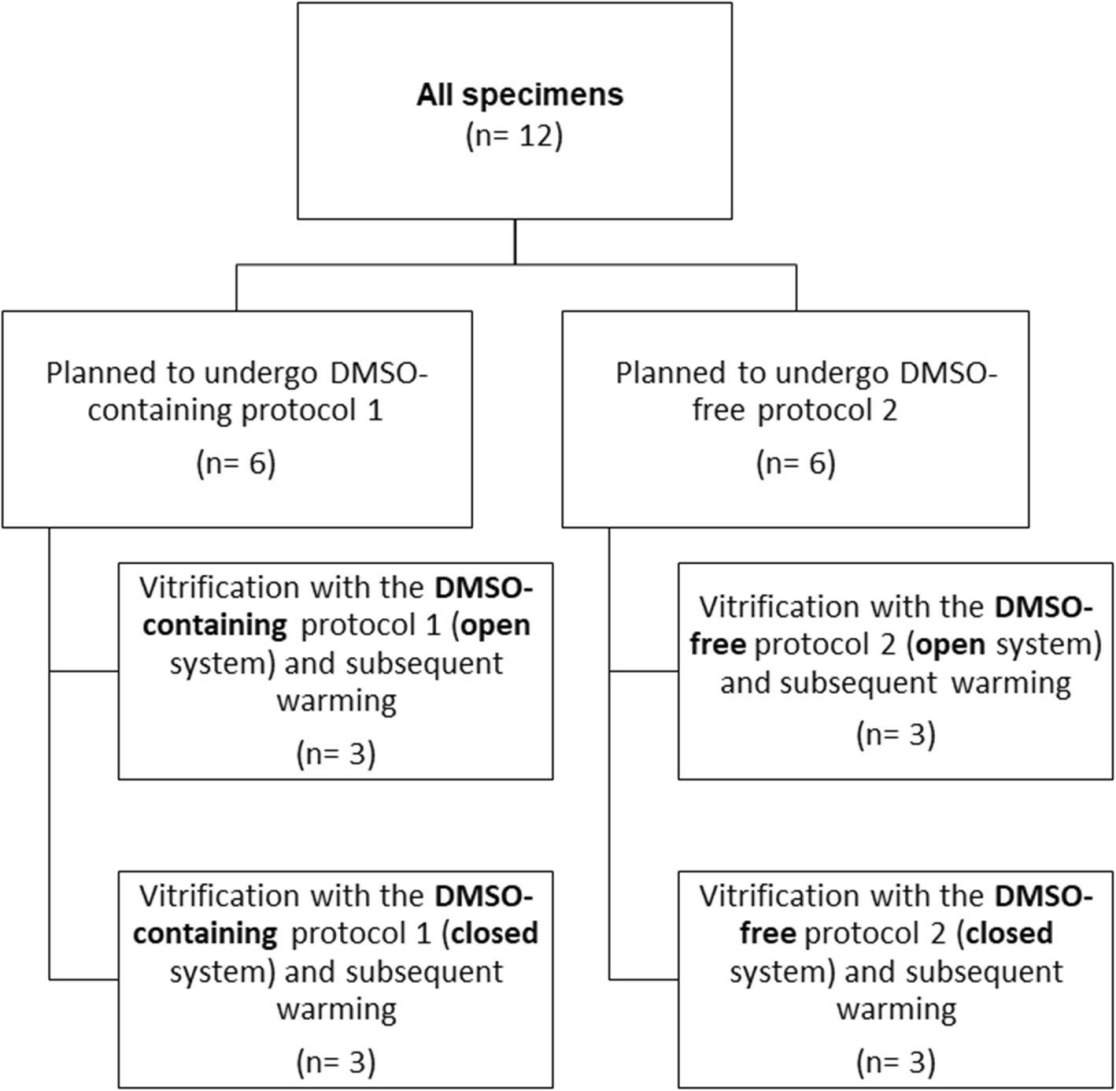
Study flow chart

In total, 12 female-to-male transgender patients, treated with androgens for a minimum of 1 year, donated their ovaries for this study, and as a result 12 ovarian samples could be obtained. Six were vitrified according to the DMSO-containing protocol 1, the other six were vitrified according to the DMSO-free protocol 2. For both protocols, an open and closed vitrification was performed (study flow chart: Fig. 1). Median age of patients enrolled to protocol 1 and protocol 2 was 22.6 (IQR 19.1-32.1) and 21.9 (IQR 18.9-29.1) years, respectively (*p =* 0.72). Basic patient characteristics are provided in Table 1.

**Table 1:** Basic patient characteristics

	DMSO-containing (protocol 1)	DMSO-free (protocol 2)	***P***-value
Age (years)	22.6 (19.1;32.1)	21.9 (18.9;29.0)	0.716
BMI (kg/m^2^)	23.2 (21.8;26.2)	23.9 (21.1;24.8)	0.780
FSH (mU/ml)	3.1 (1.8;5.7)	6.5 (3.6;7.3)	0.195
LH (mU/ml)	1.5 (0.5;5.5)	3.5 (1.0;8.7)	0.780
Estradiol (pg/ml)	39 (36;52)	39 (22;42)	0.173
Testosterone (ng/ml)	5.6 (3.2;7.6)	4.9 (4.3;6.1)	0.701
SHBG (nmol/l)	25.2 (21.7;38.2)	28.0 (21.0;46.1)	0.652
Treatment duration (months)	17 (15;33)	17 (11;18)	0.183

Values are provided as median and interquartile ranges

*BMI* Body mass index, *FSH* Follicle stimulating hormone, *LH* Luteinizing hormone, *SHBG* Sexual hormone binding globulin, *treatment duration* Duration of androgen treatment (months)

### Fluorescence activated cells sorting (FACS) analysis

**Fig. 2:**
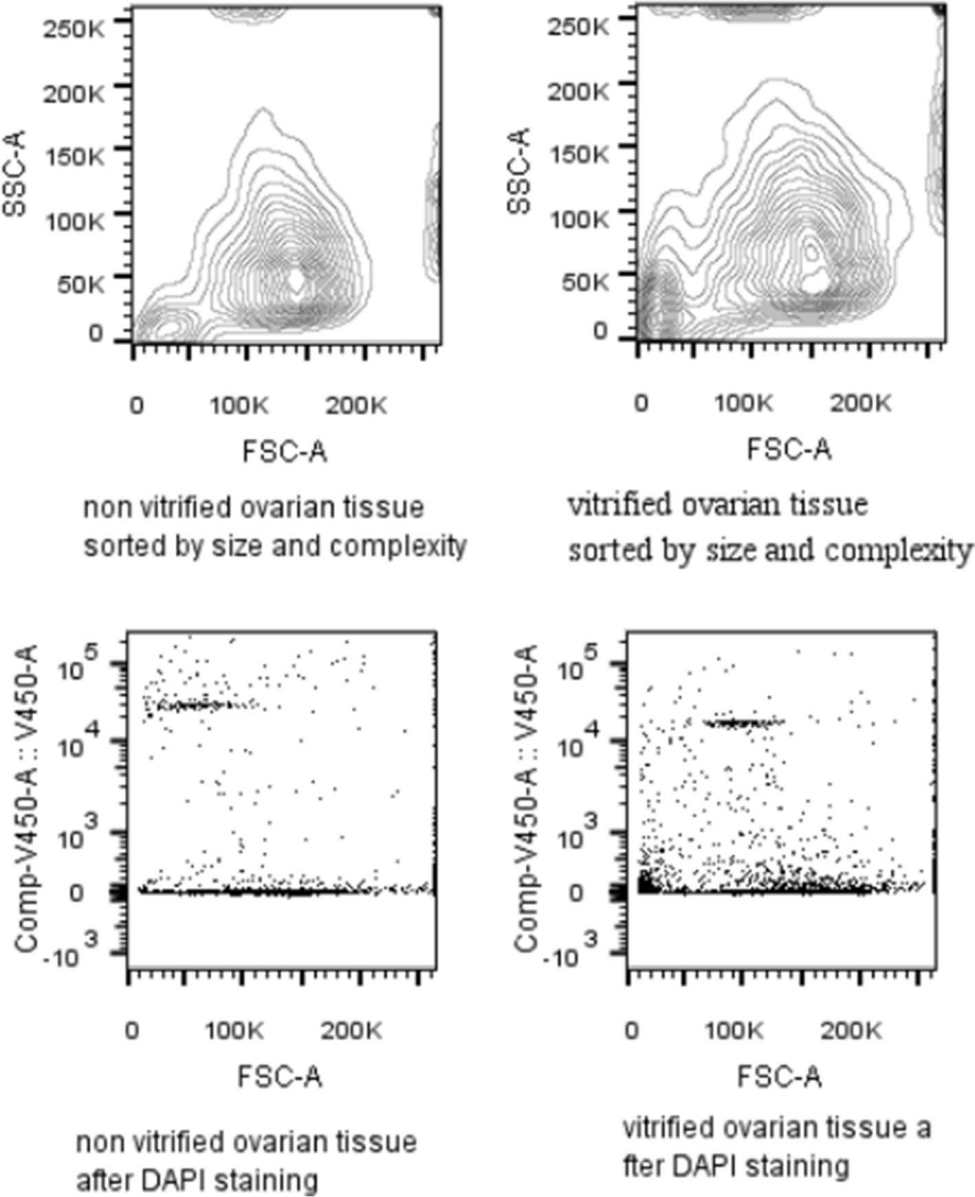
Representative example image of a Fluorescence Activated Cells Sorting (FACS) analysis of non-vitrified (left) and vitrified (right) ovarian cells after enzymatic digestion. Upper lane: Contour Plot of ovarian cells. Lower lane: Dot Plot of DAPI (4,6 Diamino-2-Phenylindole, Dihydrochloride) stained cells

Ovarian tissue consists of a variety of different cells and cell types, which in turn form different cellular structures. These structures and the cell-cell connections in such a cell network may eventually be affected during vitrification. Thus, mechanical and/or enzymatic digestion can be different for individual cell types. Figure 2 exemplarily shows that the cell populations before and after vitrification did not differ fundamentally according to our FACS analysis: their size and complexity seemed to be similar in fresh and vitrified ovarian tissue.

**Fig. 3:**
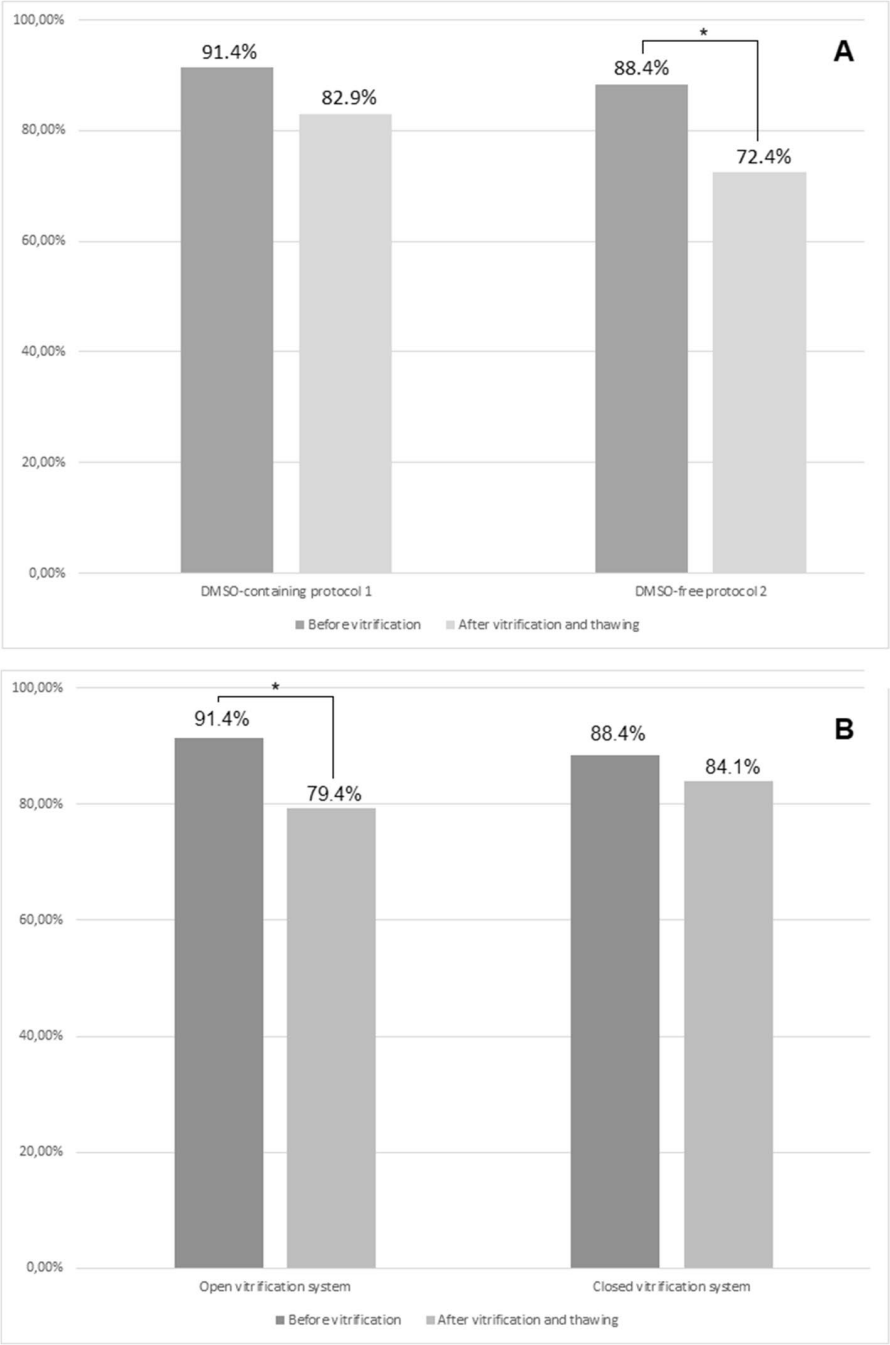
**A** Rate of viable ovarian cells before and after vitrification/warming using the DMSO-containing protocol 1 and the DMSO-free protocol 2. **B** Rate of viable ovarian cells before and after vitrification/warming using the open and the closed vitrification system. *p < 0.05

In order to obtain a control amount of viable cells from all the specimens, directly after surgical removal of ovarian tissue, the numbers of viable and non-viable cells were determined, before vitrification. In all 12 samples, regardless of the vitrification methods used, the amount of viable cells was 91.4% (IQR: 80.7-95.7) before vitrification and 80.1% (IQR: 68.8-92.1) after vitrification and subsequent warming (*p =* 0.050). Concerning the specimens intended to undergo the DMSO-containing protocol 1 and the DMSO-free protocol 2, the amount of viable cells was 91.4% (IQR: 84.5-97.0) and 88.4% (IQR: 78.1-95.1) before vitrification, respectively (*p =* 0.528; see Figs. 2 and 3, first bars). After vitrification and subsequent warming, the amount of all viable cells was 82.9% (IQR: 78.1-91.9) for protocol 1 and 72.4% (IQR: 53.9-92.4) for protocol 2 (*p =* 0.057). This decline in cell viability was significant only for the DMSO-free protocol 2 (*p =* 0.019), but not for the DMSO-containing protocol 1, (*p =* 0.093; Fig. 3).

In a next step, the open method and the closed method were compared to each other. After the vitrification and warming process, there were no differences in cell viability between the open system (median 79.4%, IQR: 59.1-90.5) and the closed system (84.1%, IQR: 69-4-92.4; *p =* 0.459). Within the subgroups of the open system, the decline in cell viability from pre- to post-vitrification was significant (*p =* 0.037), whereas this was not the case for the closed system (*p =* 0.139).

### Histological analysis

For ovarian tissue re-transplantation, and subsequent restoration of the woman’s fertility, the number of intact follicles in the tissue after freezing is a crucial parameter. Before freezing, a median number of 86 follicles (IQR 10-271) was found for the DMSO-free group, compared to a median number of 70 (IQR 13-118) in the DMSO-containing group (*p* = 0.426). Notably, a sub analysis revealed that the total number of follicles before freezing was significantly associated with age (ß = − 13.6 ± 5.8; *p* = 0.019) in a generalized linear model, whereas this was not the case for the duration of treatment. Neither of the parameters was associated with the number of defective follicles before freezing (Table 2).

**Table 2:** Generalized linear model for the prediction of the number of follicles before freezing

	Prediction of total number of all follicles		Prediction of total number of defective follicles	
	ß ± standard deviation	*p*-value	ß ± standard deviation	*p*-value
Age (years)	−13.6 ± 5.8	0.019	−0.3 ± 0.3	0.312
Duration of treatment (months)	−6.7 ± 4.7	0.153	0.1 ± 0.3	0.771

*treatment duration* Duration of androgen treatment (months)

**Fig. 4:**
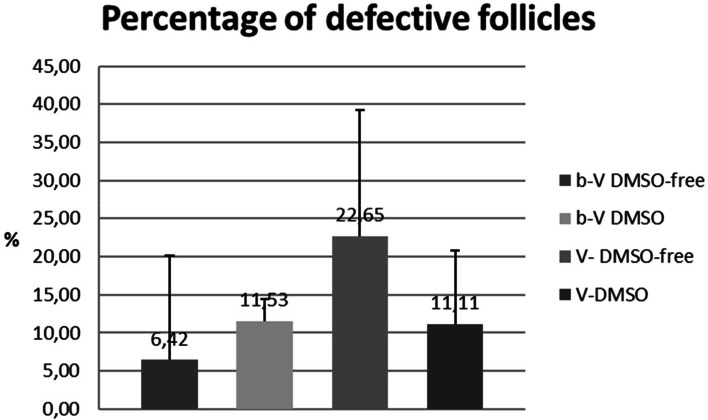
Number of defective follicles with regard to different protocols

**Fig. 5:**
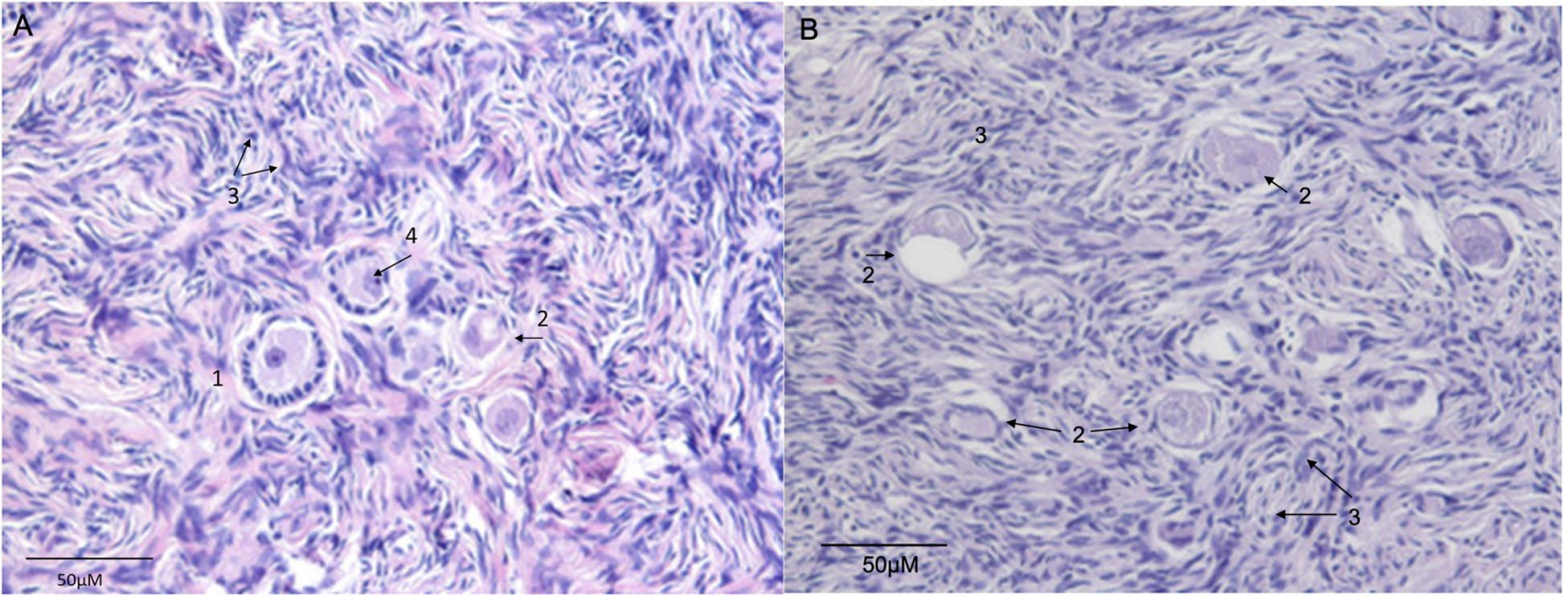
Example of morphologically normal and defective follicles. Example of HE stained ovarian histological tissue sections before (**A**) and after (**B**) vitrification showing normal (1) and atretic (2) follicles. Stroma cells (3) and Oocyte nucleus (4) were marked (Magnification 400X)

We determined the number of defective follicles in histological examinations before and after vitrification with respect to the different protocols (Fig. 4). Examples of normal and defective follicles are given in Fig. 5. Although not statistically significant, this analysis revealed that the proportion of defective follicles was greatest in the DMSO-free group (6.42% before vs. 22.65% after vitrification, *p =* 0.12). In the group vitrified with DMSO the proportion of defective follicles was 11.53% before and 11.11% after vitrification (*p =* 0.9).

Histological determination of the percentage of defective follicles after vitrification in relation to the percentage of defective follicles before vitrification. Values are given as a percentage. Bars represent means and standard deviation (SD) of 6 individual experiments. There were no significant differences between the protocols (*p* = 0.145 in ANOVA).

(b-V DMSO-free: Percentage of defective follicles before vitrification with ethylene glycol, b-V DMSO: Percentage of defective follicles before vitrification with dimethyl sulfoxide, V-DMSO-free: Percentage of defective follicles after vitrification with ethylene glycol, V-DMSO: Percentage of defective follicles after vitrification with dimethyl sulfoxide)

## Discussion

The results of our study show that in general, the majority of ovarian cells were viable after vitrification and subsequent warming despite a significant decline in the number during the process. However, what emerged was that there were significant differences depending on the protocol used.

When considering the effects of vitrification on ovarian tissue, several factors must be considered, including size of the tissue, cooling and warming rates as well as the type and concentration of cryoprotectants. Both, DMSO and ethyleneglycol were reported to have concentration-dependent toxic effects on ovarian tissue [14–16]. Depending on its concentration, DMSO decreases the cell viability and increases apoptosis and necrosis in certain cell lines [17]. Notably, at higher concentrations, it acts pro-oxidant, whereas at low concentration it acts as a radical scavenger exhibiting antioxidant activity [18]. Our results show that vitrification using a DMSO-free protocol resulted in a significant decline in the rate of viable cells after the vitrification and warming process (*p =* 0.019), whereas this was not the case when a DMSO-containing protocol was used (*p =* 0.093). Of note, this result is in accordance with our previous findings where a DMSO-containing protocol resulted in a lower number of non-viable granulosa cells compared to an ethylenglycol-containing protocol [19]. Moreover, a recent study demonstrated significant changes in microRNAs and alterations in the epigenetic landscapes of cardiac and hepatic cells after treatment with 0.1% DMSO which led the authors to conclude that DMSO was not inert and might have some impact on embryonic development [20]. In contrast, Amorim and colleagues demonstrated that a DMSO free medium containing ethyleneglycol and trehalose has no deleterious effect on follicular morphology [21]. A 2013 study concluded that vitrification solutions containing only EG without DMSO reveal similar results with respect to follicular morphology when evaluated by Transmission emission electrone microscopy (TEM) and apoptosis determined by caspase-3 immunostaining [22]..

Primordial follicles – routinely used for determining the efficiency of cryopreservation – represent more than 90% of ovarian follicles [9, 23, 24]. It must be taken into account that ovarian tissue consists of a large number of different cell types requiring different parameters to avoid ice crystal formation during the freezing process [6]. Of note, adequate preservation of stroma and vascular system is of fundamental importance due to their critical role in follicular development and restoration of gonadal function after re-transplantation. It appears therefore logically consistent, that the efficiency of a cryopreservation protocol should be evaluated not only by an analysis of primordial follicles, but also by an analysis of other cells contained in cortical ovarian tissue: with respect to our FACS analysis, the difference between non-viable cells before and after vitrification is rather small with the DMSO-containing protocol, and higher with the DMSO-free protocol.

An open vitrification system bears the risk of a transmission of infective agents, and infections following artificial reproductive technologies (ART) have already been described in animals and humans [13, 25–27]. Although none of these reported infections was ascribed to the cryopreservation technique itself, infective contamination via cryopreservation has experimentally been shown [12, 13]. Closed vitrification systems are considered controversial as they may prolong the cooling rate especially in larger tissue, which is critical in very sensitive samples. Hence, there is still a demand for a successful scientifically proven closed vitrification system in OTC [13]. Interestingly, our data show that the use of an open system was associated with a significant decline in the rate of viable cells after vitrification and warming (*p =* 0.037), which was not the case for the closed system (*p =* 0.139). Although there was no difference in final survival rates between the systems, this can be seen as a hint that open systems might be more prone to increased cell death. Our finding is in line with a recent study demonstrating that vitrification of human ovarian tissue with a closed system provides similar efficacy compared to vitrification with an open device [28]. Moreover, Terren and colleagues demonstrated that transplanted mouse ovaries after both open and closed vitrification, resulted in restored ovarian cycles, although no pregnancies were achieved with the closed system in this study [29].

A major limitation of our study is the small number of samples, with ovarian tissue donated from female-to-male transgender patients. Another limitation is the fact that we did not assess contamination rates, comparing closed and open systems. The specific effect of androgens on the viability of ovarian tissue is unknown and discussed controversially [30, 31]. Other studies indicate that a long-term androgen treatment does not seem to reduce the primordial follicle pool, and that ovaries of transmen represent an excellent source of tissue for research purposes [32, 33]. We are aware, that the viability of ovarian tissue can only be determined after reimplantation, but using FACS analysis, we were able to demonstrate the viability of different cell types, which we regard as a strength of our study. Using FACS analysis in ovarian tissue is a relatively new methodical approach, which has been only rarely used, so far [33, 34]. Evaluating ovarian tissue after vitrification by using morphological parameters and histologic analyses is the main focus of most studies, based on the assumption that morphological intact follicles are representative for the success of ovarian tissue re-transplantation. Of note, the variety of cell-types surrounding the follicle seems to play an essential role after re-transplantation of ovarian tissue as well [35, 36].

Taking into account that individual cell types might react differently on mechanical and/or enzymatic digestion, we initially showed with our FACS analysis that size (measured by forward scatter) and complexity/granularity (measured by side scatter) of the cell populations before and after vitrification show a high similarity. To the best of our knowledge, we are the first to report such results in OTC.

In conclusion, our results lend support to the hypothesis that a protocol containing DMSO results in a higher viability of ovarian cells than a protocol that uses ethylene glycol as a cryoprotective agent in vitrification. With the protocols used, the closed system revealed no significant decline in the rate of viable cells after vitrification and warming, whereas the open system did. Larger studies and the consideration of an animal model (such as a SCID mouse) for reimplantation might prove beneficial to confirm our findings.

## Materials and methods

### Study design and collection of ovaries

Donated ovaries were collected from female-to-male transgender donors during their combined gender reassignment operation, which findings have been documented and published in a previous paper and which included total laparoscopic hysterectomy, bilateral salpingo-oophorectomy and bilateral mastectomy [37].

Patients were recruited between February 2017 and December 2018. Inclusion criteria were: (i) gender-completing operation; (ii) age 18–40 years; (iii) willingness to donate ovarian tissue for research purposes.

### Ethical approval

Oral and written informed consent was obtained from all participants. The study was approved by the ethics committee of the Medical University of Vienna (EK 2240/2016), was conducted in accordance with the Declaration of Helsinki and was retrospectively registered in the Current Controlled Trials Register (NCT03649087).

### Surgical technique and tissue preparation

To ensure short ischemia times during hysterectomy and bilateral salpingo-oophorectomy, the ovarian perfusion through the infundibulo-pelvic (IP) ligament was obtained until dissection of the uterus was completed. After dissection of the IP ligament and opening of the vaginal cuff, uterus, fallopian tubes and ovaries were removed en block through the vagina. Dissection of ovarian tissue was performed on a side table directly after oophorectomy. The ovary was cut into two pieces in a petri dish using a scalpel and sterilized forceps, whereby two-thirds of the ovary was sent for histo-pathologic examination and morphological evaluation of the follicles. The remaining part of the ovary was transported in PBS at room temperature to the laboratory where the medulla was separated from the cortex under a laminar airflow, again with the use of a scalpel and sterilized forceps, by scraping it from the cortex and washing it in PBS to remove any remaining blood cells. Afterwards the cortex was cut into pieces of 10 mm × 5 -10 mm × 2 mm for subsequent vitrification.

### Vitrification and warming

Two different vitrification protocols were used, with both being performed in an open and in a closed system. For the open system, the 1.8 ml cryovial tubes (Nunc, ThermoFisher Waltham, Massachusetts, US) including the tissue pieces were closed with a lid, after immersion in liquid nitrogen; whereas for the closed system, the cryovial tube was closed directly before the immersion in liquid nitrogen. The study flow chart is provided in Fig. 1.

The first protocol contained DMSO as cryoprotective agent and was previously described by *Silber* et al. (protocol 1) [1]. The second contained ethylene glycol and propylene glycol (propane-1,2-diol) as cryoprotective agents and was purchased by ORIGIO (Origio, Måløv, Denmark (protocol 2).

Except otherwise stated, all chemicals were obtained from Sigma (Sigma Chemical Co., St Louis, USA). As warming solution, we used the MediCult Vitrification Warming (Origio, Måløv, Denmark). The composition of the warming solution can be found online at https://coopersurgical.marketport.net/MarketingZone/MZDirect/Source/65f77e06-69c4-479e-be14-9e9254b3650c (accessed on Sept, 28th 2021).

### Protocol 1

For vitrification according to the protocol previously published by *Silber* and colleagues [1] the washed pieces were equilibrated for 10 min. in equilibration solution (ES) containing 7,5% Dimethylsulfoxid (DMSO) and 10% fetal calf serum (FCS) in DMEM. Afterwards the pieces were transferred with a tweezer to the vitrification solution (VS) containing 20% ethylene glycol (EG), 20% DMSO, 0.5 M Sucrose and 10%FCS in DMEM. After 10 to 15 min of incubation, the tissue pieces were removed from the VS and transferred to a 1.8 ml tube (Nunc, ThermoFisher Waltham, Massachusetts, US) with tweezers. The tubes were then quickly immersed in liquid nitrogen using a tube holder.

For warming, the samples were removed from liquid nitrogen and 1 ml of a 37 °C pre-warmed warming solution containing 1 M sucrose and 20% FCS were added. After incubation for 1-3 min. in a water bath (37 °C) the pieces were transferred to 1 ml of a solution containing 0.5 M sucrose and 20% FCS for 3 min. After a washing step with PBS the ovarian pieces were either used for formalin fixation and histological examination or for FACS analyses.

### Protocol 2

For vitrification according to the DMSO-free protocol by *Origio*, we used the *MediCult Vitrification Cooling kit* for human oocytes, cleavage stage embryos and blastocyst and adapted the protocol for our purposes as follows: The equilibration and vitrification solutions were warmed to room temperature. The ovarian tissue pieces were equilibrated for 15 min. in equilibration solution and transferred afterwards with a tweezer to the vitrification solution (VS) for a maximum of 1 min. The tissue pieces were then removed from the VS and transferred to a 1.8 ml tube (Nunc, ThermoFisher Waltham, Massachusetts, US) with tweezers. The tubes were immersed in liquid nitrogen using a tube holder.

For warming we used the *MediCult Vitrification Warming kit* by *Origio* and adapted the protocol as follows: After removing the samples from liquid nitrogen 1 ml of a 37 °C pre-warmed warming solution was added directly into the tube, the tube was incubated in a 37 °C water bath for a maximum of 3 min. Afterwards the ovarian tissue pieces were transferred into dilution media 1 for 3 min. Then the tissue was transferred for another 3 min in dilution media 2. After washing steps with washing media and PBS the ovarian pieces were either used for formalin fixation and histological examination or for enzymatic digestion and subsequent FACS analyses.

### Enzymatic digestion with Liberase

For the enzymatic digestion with Liberase, we adapted protocols by Vanacker [38] and Dolmans [39] for our purposes: the tissue was sliced into small pieces with a scalpel and was transferred in 10 ml PBS. After addition of 215 μl of Liberase DH (2,8 Wünsch Units) the suspension was incubated for 1 h at 37 °C. The suspension was shaken every 15 min. With a pipette for additionally mechanically disruption. After the digestion step, the cell suspension was filtered through a 100 μm filter and rinsed with PBS. The cells were collected by centrifugation (300rcf) and washed twice in PBS.

### Fluorescence activated cell sorting (FACS) analysis

FACS was used due to its ability to discriminate vital cells from non-vital cells after staining with appropriate dyes: The cells were suspended in 1 ml PBS and analyzed on a BD FACSVerse Flow cytometer. DAPI (4,6 Diamino-2-Phenylindole, Dihydrochloride) was added to the samples 10 min before start of the analysis to distinguish viable and non-viable cells. The percentage of viable and non-viable cells was analyzed in the fresh tissue directly after preparation of the ovary and was used as a control for untreated (not vitrified) cells. We used *n* = 6 samples for each protocol. In every sample 10.000 events were determined using fluorescence activated cell sorting (FACS). The ratio of viable and non-viable cells was given as a percentage. Data were analyzed using a BD FACSuite V1.06 and FLOWJO software (www.flowjo.com).

### Histological analysis

Fresh and warmed ovarian pieces were fixed in 4% buffered formaldehyde and embedded in paraffin blocks. The paraffin blocks were serially cut into 4 μm sections and stained with hematoxylin and eosin. Within these sections, the number of follicles present were recorded and classified as primordial (oocyte surrounded by a single flat layer of follicle epithelial cells/pre-granulosa cells), primary (single layer of cuboidal granulosa cells), secondary (two or more layers of granulose cells, no antrum), or antral (presence of an antrum), similar to previously described methods [23, 40–42]. Morphologic evaluation of the follicles was based on examination of the integrity of the basement membrane, cellular density, presence or absence of pyknotic bodies, and integrity of the oocyte. Based on these criteria, follicles were classified as morphologically normal or abnormal.

### Statistical analysis

The statistical analyzes were performed using the SPSS 27.0 software. Data are provided as median and interquartile ranges (IQR). The data of the FACS analyzes were analyzed using ANOVA for independent samples followed by Tukey’s HSD Test. Generalized linear models were used to test for the predictive capacity of age and duration of previous treatment for the total numbers of follicles before freezing. Differences were considered statistically significant at *p <* 0.05.

## Data Availability

The datasets used and analyzed during the current study are available from the corresponding author on reasonable request.
